# Association between prolonged stress caused by COVID-19 pandemic and earthquakes and quality of life, anxiety, depression, psychoactive substances, and problematic alcohol use in adult Croatian population

**DOI:** 10.3389/fpsyt.2024.1295977

**Published:** 2024-02-29

**Authors:** Zrnka Kovačić Petrović, Tina Peraica, Mirta Blažev, Dragica Kozarić-Kovačić

**Affiliations:** ^1^ Department of Psychiatry and Psychological Medicine, University of Zagreb School of Medicine, Zagreb, Croatia; ^2^ Department of Addiction, University Hospital Vrapče, Zagreb, Croatia; ^3^ Department of Psychiatry, Referral Center for Stress-related Disorders of the Ministry of Health, University Hospital Dubrava, Zagreb, Croatia; ^4^ Department of Forensic Sciences, University of Split, Split, Croatia; ^5^ Ivo Pilar Institute of Social Sciences, Zagreb, Croatia

**Keywords:** substance abuse, alcohol drinking, prolonged stress, quality of life, mental health

## Abstract

**Background:**

The prolonged stress experience caused by the COVID-19 pandemic and two earthquakes led to increased alcohol and psychoactive substance use (PSU) accompanied by a decrease in mental wellbeing and quality of life (QoL) in the Croatian population. Our aim was to determine the relationship between alcohol and PSU and mental health outcomes including anxiety and depression, and QoL.

**Methods:**

A cross-sectional online survey conducted from September 30 to October 27, 2021, included 1,118 Croatian adults (220 men and 898 women; mean age, 35.1 ± 12.3 years) recruited through non-probabilistic convenience sampling. The survey consisted of a self-reported questionnaire on PSU, the CAGE Alcohol Questionnaire, the Hospital Anxiety Depression Scale, and the World Health Organization Quality of Life (WHOQoL)—BREF. Structural equation modeling was used to evaluate the association between PSU, problematic alcohol use (PAU), mental health outcomes, and QoL.

**Results:**

The model demonstrated a good fit and indicated that PSU increase, PAU, and anxiety and depression symptoms significantly explained all QoL domains (*p* < 0.001 for all). Both PSU increase and PAU during prolonged stress were directly associated with decreased QoL. These relationships were also indirectly mediated through increased anxiety and depression symptoms.

**Conclusion:**

These results showed the need to direct public health interventions and treatment interventions during and after long-term stress (pandemics and earthquakes) to reduce the negative impact on substance use and QoL by reducing depression and anxiety, which ultimately may contribute to better wellbeing and rapid recovery of individuals affected by prolonged stress.

## Introduction

Traumatic events, such as natural disasters, abuse, traffic accidents, or loss of loved ones, and prolonged stress may elicit different negative psychological responses and have different psychological consequences (1, 2). Pandemic-related stress may trigger alcohol drinking and substance use to ease coping with mental health challenges (3) and provide some relief from the pressure of prolonged stress (4). This can result in the misuse of psychoactive substances (PSs) or intensify addictive behaviors (5, 6). Increased consumption of sedatives, narcotics, alcohol, benzodiazepines, and other PSs was observed during the COVID-19 pandemic (7). In that period, a worldwide increase in anxiety and depression was also noted (8). The same findings were described after earthquakes (9, 10). Furthermore, decreased quality of life (QoL) associated with impaired mental health was found during the COVID-19 pandemic (11–13) and prolonged stress (pandemic and earthquakes) (14).

During the first three waves of the COVID-19 pandemic [the first, mild (mid-March to early May 2020); the second, strong (end of September 2020 to mid-February 2021, peaking in early December 2020), and the third, moderate (mid-February to early June 2021, peaking in mid-April 2021)] (15), two devastating earthquakes struck Croatia, one affecting the capital of Zagreb in March 2022 and the other Petrinja, a town 80 km SE from Zagreb, in December 2022. The earthquakes affected a large part of the population in Croatia.

The leading causes of hospitalizations for mental disorders in 2021 were substance use and depressive disorders (16). At the primary healthcare level in 2020, anxiety disorders accounted for 52.3%, mental and behavioral disorders caused by alcohol consumption accounted for 2.0%, and mental and behavioral disorders caused by psychoactive substances accounted for 1.3% of mental health disorders (17).

According to the 2022 Croatian Institute of Public Health data, the share of first-time registered persons treated for psychoactive substance abuse decreased in 2020 and 2021 in comparison with that pre-pandemic 2019, whereas the share of first-time registered persons treated for opiate use increased (18). The study performed on 2,860 Croatian adults during the first wave of the COVID-19 pandemic found that 15.9% of the respondents experienced severe to extreme depression, 10.7% severe to extreme anxiety, and 26.2% severe to extreme stress (19).

During the COVID-19 pandemic, QoL was decreased in individuals with non-treated alcohol use disorder (20) and individuals on opioid substitution therapy (21). Insufficient sleep was associated with a lower QoL score and psychoactive substance use (PSU) among Polish students (22). A significant prevalence of adolescent smoking was found in an outpatient clinic sample (23), and tobacco smoking had a negative impact on the QoL of vocational students (24).

It has been shown that increasing resilience can act as a mitigating factor against negative effects on subjective wellbeing and mental health outcomes among earthquakes in Turkey (25). Implementing tailored interventions to alleviate peritraumatic distress can lead to amelioration of pessimism by providing individuals with effective coping strategies and support systems (26). The psychological consequences of an earthquake can be mitigated by comprehensive measures through psychosocial support, social networks, and government interventions to enable individuals and communities to recover (27).

We found reports that QoL was decreased after exposure to natural disasters, like earthquakes (12, 13, 28). However, we did not find any study investigating the association between QoL and PSU after exposure to earthquakes. To the best of our knowledge, this is the first study to examine the relationship between QoL and PSU in adults who experienced the COVID-19 pandemic and earthquakes at the same time. The study also investigated the relationship between QoL and different PSU (tobacco, caffeine, alcohol, cannabinoids, cocaine, stimulants, anxiolytics, hallucinogens, opioids, volatile solvents, synthetic cathinones, synthetic cannabinoids, and others) during prolonged stress.

We carried out an online survey to determine the direct association between increased PSU and problematic alcohol use (PAU) with anxiety and depression symptoms and different QoL domains and to determine the indirect association between increased PSU and PAU and different QoL domains through anxiety and depression symptoms during the prolonged stress in the adult population.

Our hypothesis was that the lower scores on different QoL domains were directly associated with increased PSU and that PSU increase indirectly predicted QoL domains through anxiety and depression during prolonged stress.

## Methods

### Study design and recruitment of participants

This study is an extension of our earlier research on the impact of the first three COVID-19 pandemic waves and two earthquakes on the QoL of the general adult population in Croatia (14).

We created an online survey and made it available on a Google-built website from September 30 to October 17, 2021. The information about the survey and link to the survey was posted on various social media (Facebook, Twitter, LinkedIn, Google+, and Instagram) to recruit potential participants. The study purpose and aims were described on the survey’s first page, as well as the participants’ rights. The survey was anonymous and took approximately 20 min to complete.

The Ethics Committee of the University Hospital Vrapče, Zagreb, Croatia, approved the study in accordance with the Declaration of Helsinki standards.

### Participants

The survey was completed by a total of 1,286 respondents, of whom 168 were excluded (22 minors and 146 respondents who submitted incomplete questionnaires). The final sample consisted of 1,118 participants (220 men and 898 women), whose mean age was 35.1 (*SD* = 12.3) years (age range, 18–78 years). The inclusion criteria were Croatian residence during the prolonged stress experience (caused by the first three waves of the COVID-19 pandemic and the Zagreb and Petrinja earthquakes), age 18 years or more, and ability to complete the online survey without difficulties. The exclusion criteria were as follows: not understanding the Croatian language, not signing the informed consent, and missing data. The participation was voluntary, and no financial compensation was offered.

### Study measures

#### PSU during the prolonged stress

Two *ad hoc*-developed questions were used to inquire about the use of different PSs (tobacco, caffeine, alcohol, cannabinoids, cocaine, stimulants, anxiolytics, hallucinogens, opioids, volatile solvents, synthetic cathinones, synthetic cannabinoids, and others). The first question was “Did you consume any of the listed PS 4 weeks before the pandemic and earthquakes?” The second question was “Have you increased your intake of any of the 13 listed PS in the 4 weeks before your participation in the survey?” For each PS listed, the respondents could select “yes” or “no”. Based on these two questions, the increase in PSU was calculated as a raw sum of the 13 listed PS categories. The total score ranged from 0 (no increase in PSU) to 13 (increase in use of all listed PSs). A higher score indicated increased use of listed PS. Cronbach’s α in our sample was 0.71.

#### PAU during the prolonged stress

The CAGE Alcohol Questionnaire (29), a screening tool composed of four dichotomous (0 = no; 1 = yes) items, was used to collect the data on PAU. The total score ranged from 0 to 4, with a higher score indicating more severe PAU. The cut-off of 2 is widely used for PAU (30). Cronbach’s α in our sample was 0.73.

#### Anxiety and depression symptoms

We used the Hospital Anxiety Depression Scale (HADS) (31), a 14-item screening tool for the symptoms of anxiety (HADS-A) and depression (HADS-D), on a 4-point Likert scale with a range from 0 to 3 (0 = not at all, 3 = all the time). Participants were asked to assess their anxiety and depression symptoms in the week before the data collection. The Cronbach’s α in our sample were as follows: HADS = 0.91, HADS-A = 0.89, and HADS-D = 0.83. In primary care patients and the general population, Cronbach’s α values for HADS-A were reported to vary from 0.68 to 0.93 (mean 0.83) and for HADS-D from 0.67 to 0.90 (mean 0.82) (32). HADS showed test–retest reliability scores of 0.89 for the total HADS scores, 0.87 for the HADS-A subscale, and 0.81 for the HADS-D subscale in a population of family caregivers of Alzheimer’s disease patients in Croatia (33).

#### QoL during the prolonged stress

QoL was measured using the World Health Organization Quality of Life (WHOQoL)–BREF, a 26-item, 5-point Likert scale questionnaire (34, 35) commonly used to measure different QoL domains including general QoL, health satisfaction, physical and psychological health, social relationships, and environment. The WHOQoL-BREF scale instructions advised the participants to refer specifically to the week before the data collection. The QoL domain’s reliability was as follows: physical health (α = 0.81), psychological health (α = 0.89), social relationships (α = 0.70), and environment (α = 0.78).

### Data analysis

To verify the assumption of normality, the Shapiro–Wilk test was performed for all variables in the model. To determine how PSU increase and PAU predict different QoL domains directly and indirectly through anxiety and depression symptoms, a structural equation modeling (SEM) was conducted using weighted least-square means and variance adjusted (WLSMV) robust estimator, which demonstrates good performance where variables are categorical or ordinal and data non-normality is present (36, 37). In the evaluation of model-data fit according to Hu and Bentler (38) and Browne and Cudeck (39), well-known criteria were considered: chi-square *p*-value >0.05 good, chi-square and degrees of freedom ratio <3 good/<5 acceptable, comparative fit index (CFI) and the Tucker–Lewis index (TLI) >0.95 good/>0.90 acceptable, root mean square error of approximation (RMSEA), and standardized root mean square residual (SRMR) <0.08 good/<0.10 acceptable, and *p*_close_ *>*0.05 good model-data fit. For direct and indirect effects in the model, standardized β coefficients, their *p*-values, and 95% confidence intervals (CIs) were also reported.

All statistical analyses were performed using the jamovi project (version 2.3).

## Results

### Descriptive statistics and normality assumption check

Most participants (56.1%) had either a bachelor’s or master’s degree. Most were employed (62.5%), were married or cohabitating (39.1%), and did not have any children (64.8%) (**Table 1**).

**Table 1:** Descriptive information on the demographic characteristics of the participants (N = 1,118).

	Variables	*n* (%)
Age	Age *M* ± *SD* (years)	35.1 ± 12.3
Gender	Men	220 (19.7)
	Women	898 (80.3)
Educational status	Completed primary education	3 (0.3)
	Completed secondary education	196 (17.5)
	Bachelor’s or master’s degree	628 (56.1)
	Postgraduate degree	84 (7.5)
	Current student (unfinished degree)	207 (18.5)
Marital status	Single	321 (28.7)
	In a relationship	284 (25.4)
	Married or cohabitating	437 (39.1)
	Divorced	63 (5.6)
	Widowed	13 (1.2)
Parents	No	724 (64.8)
	Yes	394 (35.2)
Employment status	Unemployed	130 (11.6)
	Employed	699 (62.5)
	Retired	29 (2.6)
	Student	221 (19.8)
	Other	39 (3.5)

On average, participants did not report excessive use of PS or PAU during the prolonged stress, but they did show mild anxiety and low depression symptoms (**Table 2**). Regarding the QoL, participants mostly perceived their QoL as relatively good in all QoL domains.

**Table 2:** Descriptive statistics of variables in the model and Shapiro–Wilk test of normality (N = 1,118).

	Variables	*M* (*SD*)	*Min*	*Max*	*Skew.*	*Kurt.*	*S−W*
Substance use	PSU	0.61 (1.17)	0	13	3.51	23.94	0.57*
	PAU	0.33 (0.80)	0	4	2.61	6.12	0.46*
Anxiety and depression symptoms	HADS-A	8.31 (4.78)	0	21	0.39	−0.44	0.08*
	HADS-D	5.90 (4.38)	0	21	0.76	0.04	0.12*
QoL domains	General QoL	3.81 (0.92)	1	5	−0.44	−0.23	0.23*
	Health satisfaction	3.88 (0.99)	1	5	−0.68	−0.04	0.23*
	Physical health	15.77 (3.03)	4.57	20	−0.77	0.33	0.10*
	Psychological health	14.63 (3.50)	4	20	−0.56	−0.38	0.10*
	Social relationships	14.89 (3.69)	4	20	−0.47	−0.35	0.10*
	Environment	15.71 (2.71)	5.50	20	−0.72	0.44	0.08*

PSU, psychoactive substance use increase; PAU, problematic alcohol use; QoL, quality of life; HADS-A, Hospital Anxiety Depression Scale—Anxiety; HADS-D, Hospital Anxiety Depression Scale—Depression; Skew., skewness; Kurt., kurtosis; S−W, Shapiro−Wilk normality test.

^*^p < 0.001.

The Shapiro–Wilk test results indicated deviation of data from univariate normality regarding PSU increase, PSU, anxiety and depression symptoms scales, and QoL domains (*p* < 0.001; **Table 2**). Therefore, regarding SEM, a robust estimator for non-normality, the WLSMV estimator was applied in further analysis.

Prior to conducting SEM, a correlation analysis was performed to examine the association between variables (**Table 3**). The analysis demonstrated significant bivariate correlations among all variables (*p* < 0.001), including PSU increase, PAU, anxiety, depression symptoms, and various QoL domains.

**Table 3:** Bivariate correlations between the psychoactive substance use increase, problematic alcohol use during the prolonged stress, anxiety and depression symptoms, and QoL domains (N = 1,118).

	PAU	HADS-A	HADS-D	General QoL	Health satisfaction	Physical health	Psychol. health	Social relation.	Environ.
PSU	0.32^*^	0.26^*^	0.27^*^	−0.21^*^	−0.18^*^	−0.23^*^	−0.25^*^	−0.15^*^	−0.21^*^
PAU	1	0.21^*^	0.19^*^	−0.19^*^	−0.13^*^	−0.18^*^	−0.25^*^	−0.19^*^	−0.17^*^
HADS-A		1	0.69^*^	−0.50^*^	−0.46^*^	−0.60^*^	−0.72^*^	−0.44^*^	−0.51^*^
HADS-D			1	−0.60^*^	−0.52^*^	−0.67^*^	−0.78^*^	−0.56^*^	−0.54^*^
General QoL				1	0.63^*^	0.61^*^	0.68^*^	0.53^*^	0.64^*^
Health satisfaction					1	0.68^*^	0.57^*^	0.44^*^	0.55^*^
Physical health						1	0.73^*^	0.52^*^	0.63^*^
Psychol. health							1	0.64^*^	0.64^*^
Social relation.								1	0.55^*^

PSU, psychoactive substance use increase; PAU, problematic alcohol use; QoL, quality of life; Psychol. health, psychological health; social relation., social relationships; Environ., environment; HADS-A, Hospital Anxiety Depression Scale—Anxiety; HADS-D, Hospital Anxiety Depression Scale—Depression.

^*^p < 0.001.

### Structural equation modeling results

**Figure 1:**
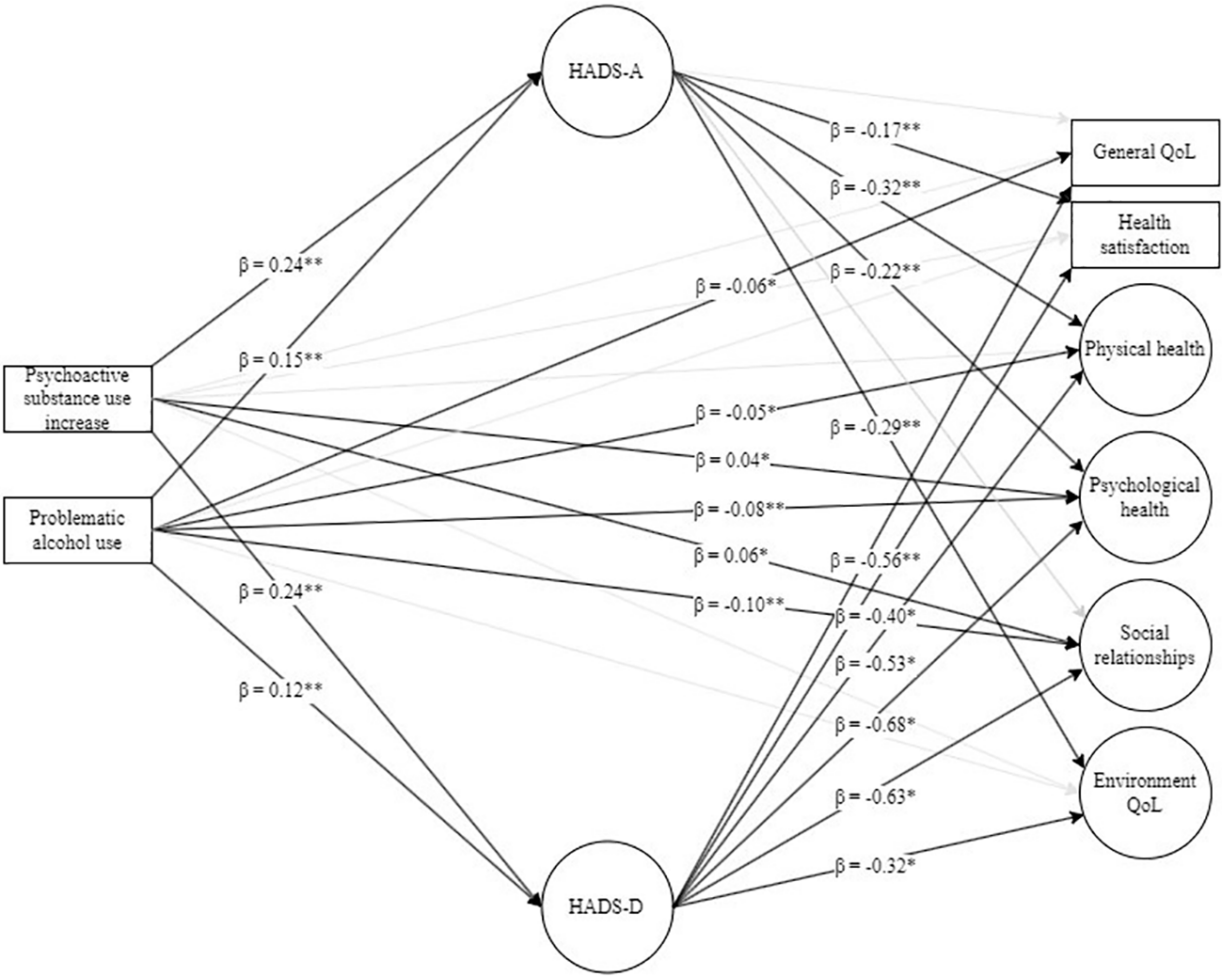
Structural equation modeling: tested model of psychoactive substance use increase and problematic alcohol use in predicting QoL domains through anxiety and depression symptoms. HADS-A, Hospital Anxiety Depression Scale—Anxiety; HADS-D, Hospital Anxiety Depression Scale—Depression; QoL, quality of life. Indicators for latent variables, as well as errors and residuals, were omitted from the figure due to clarity. Black arrows represent regression paths that are significant at *p* < 0.05, while gray arrows represent tested but non-significant regression paths. *p < 0.05, ** p < 0.01.

Results from structural equation modeling with the WLSMV estimator demonstrated a relatively good model-data fit by the Hu and Bentler and Browne and Cudeck criteria: χ^2^(658) = 2,597.85, *p* < 0.001; χ^2^/*df* = 3.95; CFI = 0.913; TLI = 0.902; RMSEA = 0.051 (90% CI [0.049, 0.053], *p*_close_ = 0.140); SRMR = 0.043. The tested model (**Figure 1**) with PSU increase, PAU, anxiety, and depression symptoms significantly explained 41.8% of general QoL, 30.7% of health satisfaction, 66.4% of physical health, 74.7% of psychological health, 49.8% of social relationships, and 36.7% of environmental variances (*p* < 0.001 for all). At the same time, PSU increase and PAU together explained 10.2% of anxiety and 8.9% of depression symptom variances.

### Direct effects of the PSU increase and PAU during the prolonged stress on anxiety and depression symptoms and QoL domains

Both increased PSU and PAU during the prolonged stress significantly predicted more severe anxiety (95% CI [0.18, 0.30] and 95% CI [0.09, 0.21]) and depression symptoms (95% CI [0.18, 0.30] and 95% CI [0.05, 0.18]), respectively (**Table 4**).

**Table 4:** Direct effects of the psychoactive substance use increase and problematic alcohol use during the prolonged stress on anxiety and depression symptoms and QoL domains (N = 1,118).

Anxiety and depression	HADS-A		HADS-D									
	β	*p*	β	*p*								
PSU	0.24**	<0.001	0.24**	<0.001								
PAU	0.15**	<0.001	0.12**	<0.001								
Model	*R* ^2^	*p*	*R* ^2^	*p*								
	0.102**	<0.001	0.089**	<0.001								
QoL domains	General QoL		Health satisfaction		Physical health		Psychological health		Social relationships		Environment	
	β	*p*	β	*p*	β	*p*	β	*p*	β	*p*	β	*p*
PSU	−0.01	0.650	−0.01	0.662	−0.01	0.648	0.04*	0.044	0.06*	0.042	−0.04	0.179
PAU	−0.06*	0.015	−0.01	0.698	−0.05*	0.034	−0.08**	<0.001	−0.10**	0.001	−0.05	0.088
HADS-A	−0.09	0.066	−0.17**	<0.001	−0.32**	<0.001	−0.22**	<0.001	−0.08	0.137	−0.29**	<0.001
HADS-D	−0.56**	<0.001	−0.40**	<0.001	−0.53**	<0.001	−0.68**	<0.001	−0.63**	<0.001	−0.32**	<0.001
Model	*R* ^2^	*p*	*R* ^2^	*p*	*R* ^2^	*p*	*R* ^2^	*p*	*R* ^2^	*p*	*R* ^2^	*p*
	0.418**	<0.001	0.307**	<0.001	0.664**	<0.001	0.747**	<0.001	0.498**	<0.001	0.367**	<0.001
Goodness-of-fit indices	χ^2^	*df*	χ^2^/*df*	*p*	CFI	TLI	RMSEA	90% CI [RMSEA]		*p*_close_	SRMR	
	2,598**	658	3.95	<0.001	0.913	0.902	0.051	[0.049, 0.053]		0.140	0–043	

PSU, psychoactive substance use increase; PAU, problematic alcohol use; QoL, quality of life; HADS-A, Hospital Anxiety Depression Scale—Anxiety; HADS-D, Hospital Anxiety Depression Scale—Depression; CFI, comparative fit index; TLI, Tucker–Lewis index; RMSEA, root mean square error of approximation; SRMR, standardized root mean square residual.

Regarding QoL domains, PSU increase significantly predicted higher satisfaction with psychological health (95% CI [0.30, 0.09]) and social relationships (95% CI [0.00, 0.12]), while it was not a significant predictor of other QoL domains (**Table 4**). At the same time, more severe PAU significantly predicted lower general QoL (95% CI [−0.11, −0.01]), satisfaction with physical health (95% CI [−0.10, −0.00]), psychological health (95% CI [−0.12, −0.04]), and social relationships (95% CI [−0.16, −0.04]), while it was not a significant predictor of health satisfaction and environmental QoL.

More severe anxiety and depression symptoms significantly predicted worse health satisfaction (95% CI [−0.27, −0.08] and 95% CI [−0.50, −0.31]), satisfaction with physical health (95% CI [−0.40, −0.23] and 95% CI [−0.61, −0.44]), psychological health (95% CI [−0.29, −0.14] and 95% CI [−0.75, −0.61]), and environment (95% CI [−0.41, −0.18] and 95% CI [−0.43, −0.21]) (**Table 4**). At the same time, more severe depression symptoms predicted lower general QoL (95% CI [−0.65, −0.47]) and satisfaction with social relationships (95% CI [−0.73, −0.53]).

### Indirect effects of the PSU increase and PAU during the prolonged stress on QoL domains through the anxiety and depression symptoms

Both PSU increase and PAU indirectly decreased satisfaction with general health, physical health, psychological health, and the environment through more severe anxiety and depression symptoms. This is indicated by negative beta coefficients (ranging from −0.03 to −0.08 for anxiety and −0.04 to −0.16 for depression) and *p*-values (all <0.05). At the same time, PSU increase and PAU both had significant indirect effects on decreased satisfaction with general QoL and social relationships through more severe depression symptoms (beta coefficients ranging from −0.07 to −0.15, with all *p*-values <0.001) (**Table 5**).

**Table 5:** Indirect effects of the psychoactive substance use increase and problematic alcohol use during the prolonged stress on QoL domains through the anxiety and depression symptoms (N = 1,118).

			General QoL		Health satisfaction		QoL domains		Psychological health		Social relationships		Environment	
							Physical health							
			β	*p*	β	*p*	β	*p*	β	*p*	β	*p*	β	*p*
PSU	→	HADS-A	−0.02	0.075	−0.04*	0.001	−0.08*	<0.001	−0.05*	<0.001	−0.02	0.144	−0.07*	<0.001
	→	HADS-D	−0.13*	<0.001	−0.10*	<0.001	−0.13*	<0.001	−0.16*	<0.001	−0.15*	<0.001	−0.08*	<0.001
PAU	→	HADS-A	−0.01	0.087	−0.03*	0.005	−0.05*	<0.001	−0.03*	<0.001	−0.01	0.156	−0.04*	0.001
	→	HADS-D	−0.07*	<0.001	−0.05*	0.001	−0.06*	<0.001	−0.08*	<0.001	−0.07*	<0.001	−0.04*	0.002

QoL, quality of life; PSU, psychoactive substance use increase; PAU, problematic alcohol use; HADS-A, Hospital Anxiety Depression Scale—Anxiety; HADS-D, Hospital Anxiety Depression Scale—Depression.

*p < 0.01.

## Discussion

In the current study, we investigated a direct association between increased PSU and PAU with symptoms of anxiety and depression and different domains of QoL and an indirect association between increased PSU and PAU and different QoL domains through anxiety and depression symptoms during long-term stress in the adult population in Croatia.

We found that PSU increase, PAU, and anxiety and depression symptoms significantly explained three-quarters of satisfaction with psychological health, more than half of satisfaction with physical health, approximately one-half of satisfaction with social relationships and general QoL, and approximately one-third of environmental QoL and health satisfaction. The tested model with PSU increase and PAU together explained 10.2% of anxiety and 8.9% of depression symptom variances.

Our results showed a positive association between the impact of prolonged stress during the COVID-19 pandemic and concomitant earthquakes on the QoL and PSU increase, PAU, and anxiety and depression symptoms. Our study participants experienced PSU increase and PAU as the impact of the prolonged stress on their decreased QoL. As alcohol is a highly available and widely accepted substance in Croatia (40), the focus of our study was on PAU measured using the CAGE Alcohol Questionnaire. The observed association of lower QoL and mental health (anxiety and depression) corresponded to the previous research findings (21, 41–44). Other studies also found decreased QoL in individuals with alcohol use disorder (20), individuals with PSU (22), individuals on opioid substitution therapy (21), and tobacco smokers (24) during the COVID-19 pandemic. An Italian study reported that a moderate psychopathological burden (anxiety and depressive symptomatology, somatization, irritability, and post-traumatic symptoms) correlated with poor QoL and low craving scores during the lockdown in individuals with substance use disorders (45). One study found that drinking patterns changed during the COVID-19 pandemic and that health-related QoL (HRQoL) decreased in the US population, indicating that individuals who reduced as well as those who increased their alcohol consumption during the pandemic might be at risk of poor HRQoL (46).

We found that the PSU increase and PAU during prolonged stress were directly associated with anxiety and depression symptoms and QoL domains. We also found indirect effects of the PSU increase and PAU during the prolonged stress on QoL domains through the anxiety and depression symptoms.

PSU increase and more severe PAU during prolonged stress were both directly associated with more severe anxiety and depression symptoms. PSU increase was significantly associated with higher satisfaction with psychological health and social relationships. This finding could be explained by the fact that PSU reduces the suffering caused by prolonged stress. Therefore, PSU could be considered a dysfunctional coping strategy for stress and trauma (3). Individuals often rationalize their behavior by downplaying the consequences of PSU for anxiety and depression associated with prolonged stress, as PSU seemingly helps them manage their social relationships (4). However, our findings show the opposite. In our study, more severe PAU significantly predicted lower satisfaction with general QoL, physical health, psychological health, and social relationships. In other words, PSU increase and PAU were positively associated with anxiety and depression, which then influenced negatively the participants’ QoL. PSU increase and PAU showed different associations with QoL domains. Although it could be expected that PSU increase negatively affected satisfaction with psychological health and social relationships, a possible explanation of our findings is that prolonged stress may trigger PSU increase to help a person cope with the stress (2). We found a direct negative influence of PAU on satisfaction with general QoL, physical health, psychological health, and social relationships. A possible explanation may be that the CAGE Alcohol Questionnaire successfully detected persons with PAU, unlike the *ad hoc*-developed questions used to identify consumption of other PS.

More severe depression symptoms were directly negatively associated with all QoL domains, while more severe anxiety and depression symptoms were directly negatively associated with lower satisfaction with general health, physical health, psychological health, and environment. A study exploring correlates of depression, QoL, and alcohol misuse during the COVID-19 pandemic also found an association between greater depression and lower QoL among international migrants in China (47).

Our findings showed an indirect association between both PSU increase and PAU and lower satisfaction with health, physical health, psychological health, and the environment through more severe anxiety and depression symptoms. Both PSU increase and PAU also had significant indirect effects on lower satisfaction with general QoL and social relationships through more severe depression symptoms.

We applied the WHOQoL-BREF (34, 35) because there are no new measurement instruments available that target domains of QoL specifically relevant in the context of PSU, including alcohol (48).

Our findings indicate direct and indirect negative associations between different QoL domains, PSU increase, PAU, and anxiety and depression symptoms. We should consider how PSU increase and alcohol consumption are connected with stressful events, such as the pandemic and earthquakes, as they may indicate underlying problems. Although our survey did not assess the reasons for changes in PSU increase and alcohol drinking from pre-pandemic levels, our results indicated that changes in PSU and alcohol consumption corresponded with changes in anxiety, depression, and QoL. We speculate that PSU increase could be a reaction to negative repercussions of prolonged stress or a way to cope with mental health challenges due to prolonged stress, or that the changes in PSU and alcohol consumption could be secondary to health effects (5, 6). Also, the associations between PSU increase, psychiatric symptoms, and QoL could be bidirectional (5, 6). Additional external factors may also have contributed to the decreased QoL. Increased time spent with family members due to social restrictions may have increased PSU and alcohol consumption and reduced QoL, and conversely, increased time spent with family and children at home may have reduced PSU and alcohol consumption and also QoL; changes in job routines could increase or decrease PSU and alcohol consumption and simultaneously reduce QoL (8). In addition, numerous changes in lifestyle during the pandemic and earthquakes could have exacerbated problems with PSU and alcohol consumption. These changes, stressors, and problems associated with PSU increase could all be related to a reduced QoL directly, as well as indirectly through anxiety and depression, leading to decreased QoL (9–14). Further research may explain the patterns found between PSU increase and alcohol consumption and QoL.

### Limitations

Our study had several limitations, as it was a cross-sectional online survey. First, the main limitation was that no causal conclusions were possible regarding the changes in the effect of the association between QoL, PSU increase, PAU severity, anxiety, and depression symptoms due to the prolonged stress experience. The study design precluded the identification of the predictive factors for increased PSU and PAU. A longitudinal study with a further survey conducted before the prolonged stress would have allowed for drawing causal conclusions about whether QoL affected PSU increase, PAU severity, anxiety, and depression; whether PSU increase, PAU severity, anxiety, and depression affected QoL; or both. Second, recall bias could also play a role. However, our study was carried out in accordance with the recommendations for conducting online research (49, 50). Online surveys have significant advantages, especially during pandemics and earthquakes, but they also have disadvantages, such as selection bias (51). Third, online surveys are more accessible to some groups of people (younger population and students) than to others (older and poorer) (52). Online research may be open to gender bias, as social networks are used more by women and online games are used more by men (53, 54). Similarly, our participants were mostly in their mid-thirties, the majority were women, and almost two-thirds had completed tertiary education. This sample composition may have had implications on our results, especially the fact that substance use disorders are generally more common among men, who were a minority in our study, and the findings cannot be applied to the general population due to selection bias.

Fourth, 10% of participants did not complete the survey. This may be due to the length of the survey (i.e., 15–20 min) and lack of financial compensation. Future studies might use shorter questionnaires and provide financial compensation to attenuate dropout rate and non-response bias. Fifth, we applied a non-validated measure of self-assessed changes in PSU (except for alcohol), which may have been interpreted differently by different participants and led to measurement errors due to self-reported bias and deviations in the real results (55). Sixth, errors in PSU measurement are possible because the questions about PSU were created by the authors (56). Seventh, we could not determine the predictive factors in relation to the QoL during the pandemic and earthquakes, which a longitudinal study could clarify. A further limitation is that only self-reported PSU and self-rating scales and no clinician-based assessments were used. As we were unable to control for confounding factors beyond those for which data were collected, such as other health conditions, the underlying causes of the observed associations remain an open question. Furthermore, there may have been other unmeasured factors influencing the association between PSU increase and alcohol consumption and QoL during prolonged stress. Possible mechanisms explaining increases or decreases in PSU and alcohol consumption include changes in physical and mental health and loneliness (57), all of which may have an impact on QoL. Finally, we were unable to extract the data on all the variables that may have contributed to PSU before and during the COVID-19 pandemic and eliminate their possible confounding effect. Future studies may use participants’ objective health-related characteristics (comorbid diseases affecting somatic or neuropsychiatric functioning, concomitant therapy, etc.) provided by healthcare professionals to obtain more robust data. However, our results are consistent with the results from cross-sectional studies in other settings, which corroborate the reliability of our findings.

Although we used the structural equation modeling for data analysis as a more robust method, caution should be exercised when interpreting the mediation findings due to the cross-sectional design.

## Conclusion

PSU or alcohol consumption may have changed as a direct result of the prolonged stress, serving as a coping mechanism, or due to environmental changes, resulting in diminished QoL.

The association between these phenomena is unclear, but data suggest that there may be a connection. The prolonged stress may have led to psychological and health effects, but the social environment may have also affected PSU increase and alcohol consumption either directly or indirectly through anxiety and depression symptoms, eventually decreasing the QoL. In future research, these effects should be disentangled to understand the relationship between the contributing factors.

## Implications

With respect to the clinical implications of our study, it showed the need to direct public health interventions and treatment interventions during and after long-term stress (pandemics and earthquakes) toward vulnerable groups to reduce the negative impact on substance use and QoL by reducing depression and anxiety.

## Data availability statement

The raw data supporting the conclusions of this article will be made available by the authors, without undue reservation.

## Ethics statement

The studies involving humans were approved by Ethics Committee of the University Hospital Vrapče, in Zagreb, Croatia (Prot. 23-1064/3-21). The studies were conducted in accordance with the local legislation and institutional requirements. The participants provided their written informed consent to participate in this study.

## Author contributions

ZK: Conceptualization, Data curation, Investigation, Methodology, Project administration, Writing – original draft. TP: Conceptualization, Data curation, Investigation, Methodology, Project administration, Writing – review & editing. MB: Formal analysis, Visualization, Writing – review & editing, Methodology. DK-K: Conceptualization, Methodology, Supervision, Writing – review & editing.
